# Enhanced Biosorption and Recovery of Copper and Zinc from Acetic Acid-Extracted Livestock Wastewater Sludge Using Baker’s Yeast

**DOI:** 10.3390/ani15060794

**Published:** 2025-03-11

**Authors:** Jung-Jeng Su, Kuang-Wei Yen, Wei-Chen Chen

**Affiliations:** 1 Department of Animal Science and Technology, National Taiwan University, Taipei 10673, Taiwan; 2 Bioenergy Research Center, College of Bio-Resources and Agriculture, National Taiwan University, Taipei 10617, Taiwan; 3 Agricultural Net-Zero Carbon Technology and Management Innovation Research Center, College of Bio-Resources and Agriculture, National Taiwan University, Taipei 10617, Taiwan

**Keywords:** livestock sludge, acetic acid extraction, baker’s yeast, biosorption, zinc, copper

## Abstract

This study aims to develop a novel use of baker’s yeast in biosorption as a sustainable metal recovery process for cost-effective and practical applications in recovering copper and zinc from waste gravity-thickened sludge generated at livestock wastewater treatment facilities. The supernatant of the acid-extracted product was separated from the residues through centrifugation. To ensure cost efficiency, the supernatant was treated with 2N acetic acid for 24 h, with the addition of hydrogen peroxide, and used for the biosorption experiments. The filtrated supernatant was adjusted to various pH values (4.5, 5.0, and 5.5) to explore the effects of acidity on the subsequent biosorption of extracted zinc and copper by baker’s yeast. A diluted molasses solution was added to the filtrate as a carbon source to support yeast growth during the 4 h biosorption experiments. The results revealed that the removal efficiency of zinc from the filtrate by baker’s yeast was 97.3%, while the removal efficiency for copper was about 48.8% at pH 5.5 with a reaction time of 4 h. In summary, this combined approach is expected to reduce and recycle heavy metals in livestock sludge. Acetic acid with hydrogen peroxide can extract copper and zinc from the sludge, and baker’s yeast can absorb both metals from the filtrate at pH 5.5 in a 4 h reaction time. This technological innovation has the potential to transform waste management practices in the livestock industry, contributing to resource recovery and environmental sustainability.

## Simple Summary

This study explores biotechnology for copper and zinc recovery from livestock wastewater sludge. Acid-extracted supernatant was treated with 2N acetic acid and hydrogen peroxide for 24 h. The supernatant was then adjusted to pH 4.5, 5.0, and 5.5, and used for biosorption with baker’s yeast in a 4 h experiment. Molasses supported yeast growth. Results showed that yeast removed over 90% of zinc and around 50% of copper at pH 5.5. This method could reduce and recycle heavy metals, extracting them with acetic acid and hydrogen peroxide, and using yeast for biosorption at pH 5.5 in a 4 h reaction time.

## 1. Introduction

Copper is commonly added to piglet feed in quantities ranging from 250 to 800 mg/kg, with higher amounts (>250 mg/kg) shown to enhance growth and improve feed efficiency. Despite these benefits, approximately 90% of the supplemented copper is excreted, leading to potential soil accumulation and pollution. Research indicates that fungi possess the ability to biosorb copper [1]. Excessive addition of copper and zinc can accumulate in the sludge of the piggery wastewater treatment system [2]. Our previous study applied environment-friendly approaches to extract copper and zinc from the gravity-thickened sludge using acetic acid instead of nitric acid or sulfuric acid. After acidic sludge extraction, some metals remain in the supernatant of the acidic sludge mixture, which might be recovered using biosorption approaches. The shortcomings of existing technologies, such as reliance on strong acids, high costs, environmental impact, and inefficiency in metal recovery, necessitate the development of more sustainable and efficient methods. The combination of acetic acid and hydrogen peroxide presents an innovative solution to these challenges. This method offers an environmentally friendly, cost-effective, and highly efficient approach to recovering copper and zinc from waste sludge. It not only reduces the environmental burden but also maximizes resource recovery, aligning with sustainable and eco-friendly practices in wastewater treatment. Thus, this study is crucial in addressing the environmental challenges posed by the accumulation of copper and zinc in wastewater sludge. It offers a novel, environmentally friendly approach to metal recovery. The innovative use of acetic acid and hydrogen peroxide, combined with biosorption using baker’s yeast, provides a sustainable and efficient solution that has the potential to transform waste management practices in the livestock industry.

Biosorption refers to a collection of physical, chemical, and metabolism-independent processes such as absorption, adsorption, ion exchange, surface complexation, and precipitation. These processes are primarily used to remove or recover substances from solutions utilizing living or dead microorganisms, algae, plants, and industrial/agricultural wastes [3]. Biosorption is categorized into bio-adsorption, bio-absorption, and bioaccumulation, employing biological materials as adsorbents [4]. Absorption involves the movement of a substance from one state to another, such as when a liquid is absorbed by a solid or when a gas dissolves in water. In contrast, adsorption is characterized by the physical attachment or bonding of ions and molecules to the surface of another molecule. Some studies further distinguish biosorption into active and passive processes. Passive biosorption is a metabolism-independent process, whereas active biosorption encompasses bioaccumulation by living cells and other interactions using biological materials [3]. In summary, bio-absorption involves the internalization of heavy metals into the yeast cells and requires live cells, while bio-adsorption involves the surface binding of metals and can occur in both live and dead cells.

Materials for biosorption include microbial mass (bacteria, cyanobacteria, fungi, yeasts, algae), industrial/agricultural wastes, natural residues, and other types (chitosan, cellulose). These materials have affinities for pollutants, with preferences for inexpensive, efficient bio-adsorbents. The mechanisms are complex and not fully understood. Passive transport involves electrostatic interactions with cell surface functional groups, leading to heavy metal complexation and precipitation [5,6,7]. Some metals are reduced to less toxic forms by cell surface enzymes, a protection mechanism seen with uranium, chromium, and cadmium. Active transport involves ion exchange and diffusion, forming precipitates or metalloproteins inside cells.

Yeast is easy to culture on a large scale and has high biomass production [8]. It is widely available as a by-product in food and beverage industries and is generally regarded as safe (GRAS). Studies show yeast tolerates heavy metals like copper, zinc, and manganese, promoting alcohol production at suitable concentrations [9]. It serves as an excellent model for metal ion removal research [10]. Various studies have used different yeast types for distinct purposes including dead vs. living cells (understanding mechanisms by comparing live and heat-sterilized yeast) [5], immobilized vs. free cells (practical applications and ease of use) [11], and pretreated cells (using physicochemical methods and industrial waste-derived yeast) [12,13]. Comparing different yeast cells helps understand possible biosorption mechanisms. For instance, Wang et al. (2017) found live yeast could not utilize radioactive uranium, precipitating it through cell-secreted phosphates. Heat-sterilized cells increased the contact surface area with uranium ions, enhancing adsorption capacity [5].

Metals are classified into hard (e.g., sodium), soft (e.g., mercury), and transition metals (e.g., zinc). Soft metals are more toxic and bond easily with nitrogen or sulfur ligands. Transition metals support biochemical reactions. Other ions in wastewater can hinder target metal adsorption. Alcohol-treated yeast shows higher biosorption for lead, copper, and cadmium without competing ions [14]. Immobilization techniques are often used on dead yeast cells, making them convenient for practical applications [11]. The study by Kordialik-Bogacka (2011) discusses the differences in biosorption capacity between live and dead yeast cells, as well as their ability to survive in extreme environments. It highlights that dead cells generally have better biosorption capacity and can survive extreme pH environments and high metal ion concentrations [15]. Combining live and dead yeast cells may offer flexibility and potential for future applications.

Biosorption typically reaches equilibrium within hours for metals like copper and zinc [16], sometimes as quickly as 10 min [17]. Short contact times may only show passive adsorption, and missing metal accumulation in cell matrices [15]. Higher metabolic rates in growth phases improve biosorption [16]. pH significantly influences biosorption, affecting functional group dissociation and wastewater reactions [18]. The biosorption capacity of copper generally increases with pH but excessive pH may cause premature metal precipitation. Based on the study by Göksungur, Üren, and Güvenc (2003), the optimal pH range for biosorption of copper ions by caustic-treated waste baker’s yeast biomass is around pH 4.0 [14]. This is where the highest copper uptake was observed. For precipitation, the pH threshold is typically higher, often above pH 7.0, where metal hydroxides start to precipitate out of the solution. However, the specific pH for precipitation can vary depending on the metal and the solution conditions [14].

Temperature impacts biosorption, indicating ion exchange mechanisms. Ali and Alrafai’s study showed that the biosorption process can exhibit both exothermic and endothermic characteristics depending on specific conditions and materials used. [16]. Optimal biosorption for metals like lead occurred at 25 °C [19]. Yeast in the lag or early growth phase exhibits higher biosorption capacity. Younger cells absorb metals better due to higher metabolic activity and detoxification mechanisms [20]. Additionally, increasing biomass concentration enhances removal efficiency by providing more surface area for metal interaction. However, metal adsorption per unit cell may decrease at higher concentrations. Dead cells show high adsorption per unit at low concentrations due to higher contact probability [12].

For some applicable studies, Göksungur et al. found that alkaline pretreatment significantly enhanced the biosorption capacity of waste baker’s yeast for copper ions, removing 60.1% of copper ions at pH 4.0 [14]. Farhan and Khadom reported optimal pH values of 6.0, 5.5, and 2.5 for lead, cadmium, and chromium adsorption, respectively, while zinc and copper favored pH 5.5, with over 95% removal efficiency [21]. De Rossi et al. used lyophilized yeast pretreated with 0.5 N NaOH, achieving a 99.7% removal efficiency for Cr (VI) at pH 5.0 (90 mg/L, 3 h) [13]. Hadiani et al. and Ghorbani et al. used response surface methodology, optimizing conditions for lead and cadmium removal [22,23]. Optimal conditions resulted in 70.3% and 76.2% removal of lead and cadmium, respectively [22], and a 6.7 mg/g adsorption capacity for cadmium [23]. This study aims to establish the optimal operation parameters for recycling copper and zinc from acidic extraction of waste sludge from livestock wastewater treatment facilities as a feed supplement.

## 2. Materials and Methods

### 2.1. Collection and Preparation of the Livestock Sludge

**Figure 1:**
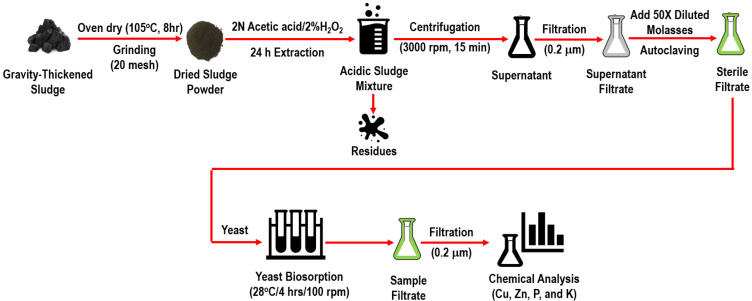
Schematic representation of acidic extraction and yeast biosorption for copper and zinc recovery from livestock sludge.

The sludge samples were collected from the sludge gravity concentrator at the National Taiwan University (NTU) livestock farm in Taipei City. The primary source of the sludge was a piggery wastewater treatment facility, processed through anaerobic digestion and activated sludge treatment. The samples were dehydrated in an oven at 105 °C for 8 h and then ground to a particle size of 20 mesh (approximately 0.84 mm) for further analysis (Figure 1).

### 2.2. Preparation of the Baker’s Yeast

A commercially available quick-rise yeast (Sunright Foods Co., Ltd., New Taipei City, Taiwan) was applied for this study, and its ingredients contain yeast and sorbitan monostearate (emulsifier). The yeast is first cultured in a yeast extract peptone dextrose (YPD) broth (Gibco™, Thermo Fisher Scientific Inc., Waltham, MA, USA) at 28 °C for 24 h [24]. The YPD broth contains (g/L) yeast extract (10), Bacto^®^ peptone (20), and dextrose (20). After culturing, the yeast is streaked on plates and purified three times, followed by Gram staining and microscopic examination to check for any bacterial contamination. The yeast culture is then stored in a slant medium at 4 °C. One day before use, it is inoculated into a YPD broth and cultured at 28 °C for 24 h. Finally, the optical density (O.D.) value is measured to analyze the concentration of the yeast culture.

Adding molasses to yeast biosorption processes can enhance the removal of copper and zinc from sludge samples. Molasses serve as a carbon source, providing essential nutrients that promote the growth and metabolic activity of yeast cells, thereby improving the biosorption capacity of the yeast [25]. In a 50-times diluted molasses solution (100% sugarcane molasses, 78 Brix, Taiwan Sugar Co., Tainan, Taiwan; pH = 5.01 ± 0.01), 0.87 ± 0.23 mg/kg of copper was detected, while zinc and chromium were not detected in multiple tests. The diluted molasses solution also contained small amounts of phosphorus (0.083 ± 0.021 mg/kg) and potassium (60.0 ± 1.62 mg/kg). Brix represents the number of grams of sucrose dissolved in 100 g of water solution at 20 °C.

### 2.3. Extraction of Copper and Zinc from the Sludge by Acetic Acid and Hydrogen Peroxide

A summary of previous experimental results indicates that the highest overall removal efficiency of zinc was achieved in two groups: those treated with 2N acetic acid for 24 h with the addition of H_2_O_2_ (2%, *v*/*v*) and those treated with 4N acetic acid for 48 h. However, the highest removal efficiency of copper was observed only in the group treated with 4N acetic acid for 48 h [2]. Regarding cost-effectiveness, the study employed the treatment group using 2N acetic acid for 24 h with the addition of 2% H_2_O_2_. This approach used acetic acid at half the concentration of the 4N group, reducing costs by approximately 50%. Additionally, using 2N acetic acid alleviated potential irritation risks associated with the strong pungent odor of the higher 4N concentration, which could affect the eyes and respiratory system during prolonged, large-scale operations.

Dried sludge powder (7.5 g) was mixed with 150 mL of acetic acid (H_3_COOH, Fisher Scientific, Loughborough, UK) in a 250 mL beaker. Then, 15 mL of hydrogen peroxide (H_2_O_2_, ECHO Chemical Co., Ltd., Toufen, Taiwan) was added. The mixture, covered with paraffin, was placed on a hot plate and stirred at 200 rpm for 24 h under ambient conditions to facilitate the acidic extraction. After the acidic extraction process was complete, the acidic sludge mixture was transferred to 50 mL centrifuge tubes and centrifuged at 3000 rpm for 15 min (Figure 1). The supernatant of the acidic sludge mixture was then transferred to clean 50 mL capped centrifuge tubes for yeast biosorption study and quantitative analysis of copper and zinc using Flame Atomic Absorption Spectroscopy (AAS). Based on the experimental results of our previous study, the optimal parameters for acidic copper and zinc extraction from the livestock sludge samples were studied under the conditions of using 2N acetic acid with 2% H_2_O_2_ for 24 h, which can remove about 40% of Cu and 70% of Zn [2]. Therefore, for subsequent experiments, the recommended treatment method is to add 2% H_2_O_2_ to the 2N acetic acid solution and apply this treatment for 24 h. In the yeast biosorption experiments, the supernatant of 2N acetic acid with the addition of 2% H_2_O_2_ for 24 h was employed as the basal medium of yeast biosorption. The supernatant of the acidic sludge mixture exhibited a pH of approximately 3.18 ± 0.24, a conductivity of 4.85 ± 0.37 mS/cm, a copper concentration of 4.49 ± 0.39 mg/kg, a zinc concentration of 45.3 ± 6.7 mg/kg, a potassium ion concentration of 53.5 ± 7.4 mg/kg, and a phosphorus concentration of 10.7 ± 1.6 mg/kg. Before the biosorption experiment, the pH of the supernatant with diluted molasses solution was calibrated using 1N sodium hydroxide.

### 2.4. Biosorption of Copper and Zinc from the Supernatant of Acidic Sludge Mixture

This experiment aims to examine the impact of different pH levels and inoculation ratios on the efficiency of copper and zinc removal via yeast biosorption from the supernatant of acidic waste solutions derived from the acid extraction of gravity-thickened sludge. Based on the previous experimental results of some studies, the optimal pH for biosorption was about 4 to 6 [21,25,26]. Table 1 shows the experimental conditions for the biosorption of metals from supernatant post-acidic sludge extraction in 125 mL Erlenmeyer Flasks. The supernatant of the acidic waste solutions at different pH levels (4.5, 5.0, and 5.5) and varying inoculation ratios (*v*/*v*) (2.5%, 5.0%, and 10%) to assess the biosorption efficiency of yeast for copper and zinc (Table 1).

The supernatant obtained after centrifugation (3000 rpm) of the sludge mixture treated with 2N acetic acid and 2% hydrogen peroxide for 24 h will first be filtered using filter paper (pore size = 0.2 μm) (Figure 1). Then, the pH of the filtrate will be adjusted to 4.5, 5.0, and 5.5 using 1N sodium hydroxide. A total of 45 mL of this solution will be taken, and 2.5 mL of the 50× diluted molasses solution will be added as a carbon source for yeast (Table 1). The solution will be placed in 125 mL Erlenmeyer flasks and autoclaved to avoid contamination.

The pH adjustments will be made using a 1N sodium hydroxide solution. The yeast suspension concentration is 3.59 mg/L, with an inoculation ratio of 5%. For the inoculation ratio experiment, the pH is set at 5.5, and the yeast biosorbent concentration at 2.05 mg/L (Table 1). Biosorption will be conducted in an orbital shaker (Shakers, Orbital Shaking, D9FF-S101, FIRSTEK, New Taipei City, Taiwan) at 28 °C and 100 rpm (Figure 1). The differences in copper and zinc concentrations before and after biosorption will be compared.

Before biosorption, the optical density (O.D.) value of the yeast culture grown the previous day will be measured to determine the concentration. A total of 2.5 mL (5%) of yeast will be inoculated into the freshly sterilized solution, making the total reaction volume of 50 mL (Table 1). This will be incubated in a shaker at 28 °C and 100 rpm for 4 h. After incubation, the mixture will be filtered using 0.2 µm cellulose nitrate membrane filters (Sartorius Stedim Biotech S.A., Göttingen, Germany) with a vacuum filtration flask. The filtered liquid will be collected in 50 mL centrifuge tubes for further analysis. The concentrations of copper and zinc in the filtrate will be determined using an atomic absorption spectrometer. The differences before and after biosorption will be compared to calculate the removal efficiency. Ion chromatography will be used to analyze the ion concentration changes before and after biosorption, inferring the ions required or interfered with during the yeast biosorption process.

### 2.5. Quantitative Analysis of Heavy Metals

Crucibles were rinsed with de-ionized water and then heated at 105 °C and put into the ashing furnace at 600 °C for 2 to 4 h. The sludge samples were ashed by placing them into the ashing furnace at 600 °C for 6 to 8 h in triplicates. The samples were mixed and heated with 5 mL 3N HCl (Fisher Scientific, Loughborough, UK) until the solid samples were completely dissolved in HCl solution. When the mixture of samples and HCl was cooled down to room temperature, the mixture was filtered with filter paper (Advantec No.1 125 mm, Toyo Roshi Kaisha, Ltd., Tokyo, Japan) and prepared to 100 mL using a 100 mL volumetric flask for further analysis of heavy metals by a flame atomic absorption spectrometer (AAnalyst 200, PerkinElmer, Inc., Waltham, MA, USA) [2].

### 2.6. Analysis of Liquid Samples

Liquid samples were filtered, and the filtrates were analyzed for anions and cations using ion chromatography (or ion-exchange chromatography) (Metrohn ion analysis; Metrohn AG, Herisau, Switzerland) [2]. The electrical conductivity of liquid samples was determined using a conductivity meter (ExStik EC500, EXTECH Instrument, FLIR Commercial Systems, Washington, DC, USA). The pH of liquid samples was determined using a pH meter (PH200, CLEAN Instruments Co., Ltd., New Taipei City, Taiwan) after calibration with standard solutions.

### 2.7. Yeast Concentration (Dry Weight) Detection

A cellulose nitrate membrane filter (Sartorius Stedim Biotech S.A., Göttingen, Germany) was placed on a filtration funnel. The suction device was activated and rinsed with at least 20 mL of deionized water continuously three times, continuing to draw out the moisture on the filter paper until completely removed. The filter paper was removed, placed on an aluminum dish, and dried at 60 °C for 2 h. After drying, it was placed in a desiccator to cool for 30 min to room temperature and then weighed. The above steps were repeated until the weight difference between consecutive measurements was within 0.5 mg. This gave the filter weight (B). The weighed filter paper was placed back on the filtration funnel. An appropriate amount of water sample was applied to the filter paper and rinsed with at least 20 mL of deionized water continuously three times, continuing to draw out the moisture on the filter paper until completely removed. The filter paper was removed, placed on an aluminum dish, and dried at 60 °C for 2 h. After drying, it was placed in a desiccator to cool for 30 min to room temperature and then weighed. The above steps were repeated until the weight difference between consecutive measurements was within 0.5 mg. This gave the dry yeast and filter weight (A). The final solid weight of the sample was 2.5 to 200 mg, and the amount of sample applied was adjusted accordingly.

Each sample was measured at least three times, and the results are expressed as the mean value ± standard deviation, with the unit in mg/L. The calculation for yeast concentration is as follows:

$$\mathrm{Yeast}\;\mathrm{Concentration}\;(\mathrm{mg}/L)\;=\;\frac{A-B}{V}$$

where the following is defined:

A: Dry yeast and filter weight (g);

B: Filter weight (g);

V: Sample volume (L).

### 2.8. Statistical Analysis

For the test of yeast biosorption, statistical analysis was conducted using a one-way analysis of variance (One-way ANOVA) to examine the effect of pH value and yeast inoculation ratio on biological adsorption. Post hoc comparisons were performed using Tukey’s HSD test. The removal efficiency percentage data were angle-transformed and compared in pairs if a significant difference was achieved. The figures and tables are based on means and standard errors (SE) and significant differences are marked when *p* < 0.05.

## 3. Results and Discussion

### 3.1. Impact of Various pH Values on Biosorption of Yeast

**Figure 2:**
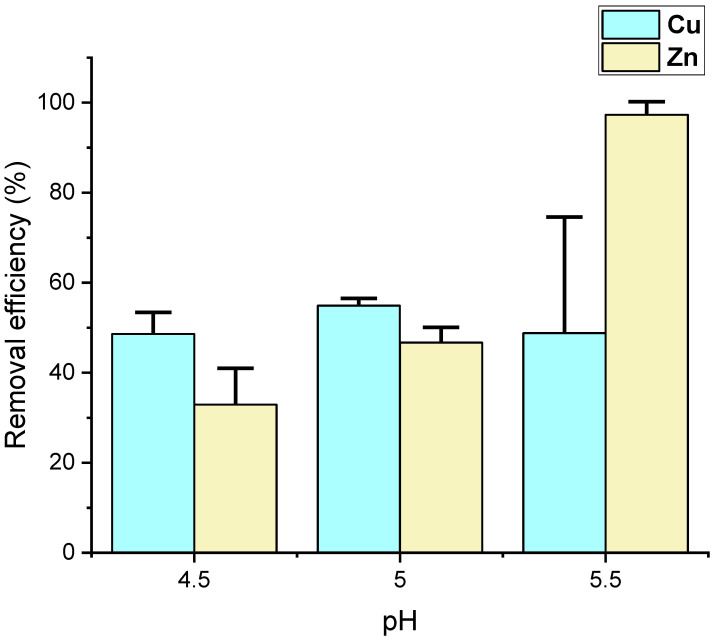
Effect of varying pH levels on the removal efficiency of copper (Cu) and zinc (Zn) by yeast biosorption from the filtrated supernatant. Error bars indicate standard deviations.

The effect of pH changes on the biosorption capabilities of yeast was investigated. Before biosorption, 1N sodium hydroxide was used to adjust the pH into three groups: 4.5, 5.0, and 5.5. The yeast’s efficiency in removing copper from the supernatant with diluted molasses showed no significant difference among the three pH groups (Figure 2 and Table 2). The highest copper removal efficiency was observed at pH 5.0, at approximately 54.9 ± 1.6%. However, no significant differences were found at the 5% level among the three groups. The removal efficiency of copper for pH 5.5 and pH 4.5 were 48.8 ± 25.8% and 48.6 ± 4.8%, respectively.

In contrast, the zinc removal efficiency of yeast increased significantly with increasing pH values. At pH 5.5, the zinc removal efficiency was 97.3 ± 2.9%, significantly higher than the zinc removal efficiency at pH 5.0 (46.7 ± 3.4%) and pH 4.5 (32.9 ± 8.1%) (*p* < 0.05) (Figure 2 and Table 2).

**Figure 3:**
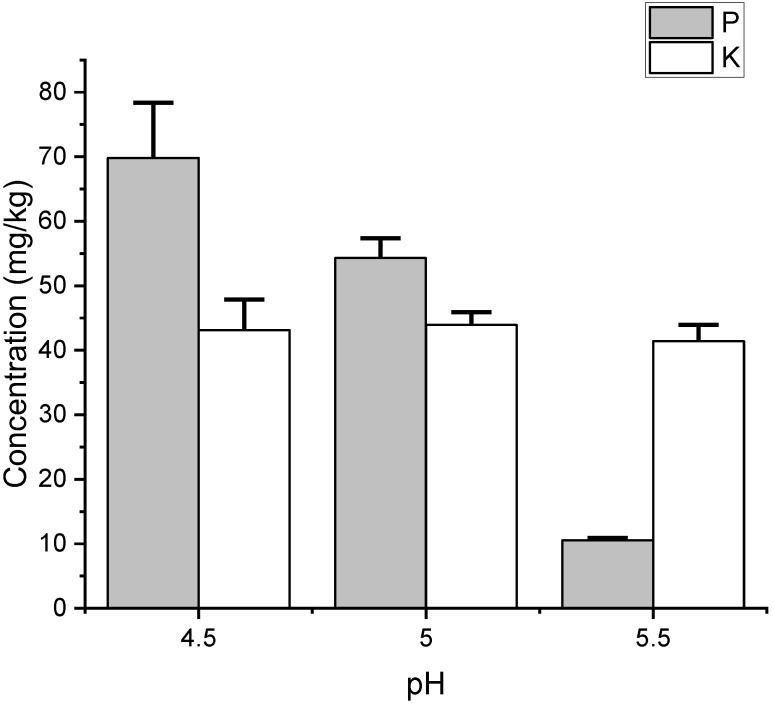
The remaining concentrations of phosphorus (P) and potassium (K) in the filtrated supernatant were evaluated at different pH values (4.5, 5, and 5.5) following yeast biosorption. The concentrations are expressed in mg/kg, with phosphorus represented by grey bars and potassium by white bars. Error bars indicate the standard deviation of the measurements.

Regarding changes in phosphorus and potassium concentrations, the initial phosphorus concentrations in the sets of pH 4.5, 5.0, and 5.5 were 72.3, 55.6, and 10.9 mg/kg, respectively. The initial potassium concentrations in the sets of pH 4.5, 5.0, and 5.5 were 44.7, 46.5, and 40.8 mg/kg, respectively (Figure 3 and Table 2). After biosorption, the phosphorus concentrations in the sets of pH 4.5, 5.0, and 5.5 were 69.8 ± 8.6, 54.3 ± 3.1, and 10.5 ± 0.4 mg/kg, respectively (*p* < 0.05). The potassium concentrations in the sets of pH 4.5, 5.0, and 5.5 after biosorption were 43.1 ± 4.7, 43.9 ± 2.0, and 41.4 ± 2.6 mg/kg, respectively (Figure 3 and Table 2).

Previous studies have shown that pH affects the dissociation ability of functional groups on the yeast surface as well as the chemical form of heavy metals in solution [12]. A lower pH can increase the concentration of hydrogen ions, which are positively charged like the heavy metals in wastewater. A higher hydrogen ion concentration can affect the electrostatic interactions between heavy metals and the cell surface, thus impacting biosorption efficiency. Generally, metal removal efficiency improves with increasing pH, though not in a linear manner. If the pH is too high, heavy metals may precipitate out of the wastewater, making them unavailable for biosorption by yeast [12].

Additionally, heavy metals are often removed from complex wastewater systems through neutralization/precipitation [27]. Most hydroxides, carbonates, and sulfides have very low solubility, and the solubility of copper and zinc decreases as pH increases. Between pH 4.5 and 5.5, zinc hydroxide has higher solubility than zinc carbonate. However, actual solubility differs from theoretical values due to coprecipitation, sometimes resulting in lower solubility than calculated. The amount of solubility and the form of metal complexes present in wastewater also affect the yeast’s biosorption efficiency.

Research by Özer and Özer indicated that at low pH, the functional groups on the yeast cell wall predominantly bind with hydrogen ions, creating repulsion against heavy metals. At pH 5.0, divalent metal ions more easily bind with negatively charged functional groups on the yeast. However, other studies found that yeast biosorption for chromium is more effective at pH 2.0, possibly due to the presence of positively charged hydrogen ions aiding the interaction with hexavalent chromium, though the detailed mechanism remains unclear [19].

For different pH treatment groups, there were no significant differences in the removal efficiency of phosphorus and potassium, both being below 10%. Results indicate that yeast uses trace amounts of phosphorus and potassium from wastewater for cell growth, without significant adsorption. This suggests that live yeast cells exhibit selectivity in absorbing elements.

### 3.2. Effect of Different Inoculum Ratios of Yeast on the Efficiency of Biosorption

**Figure 4:**
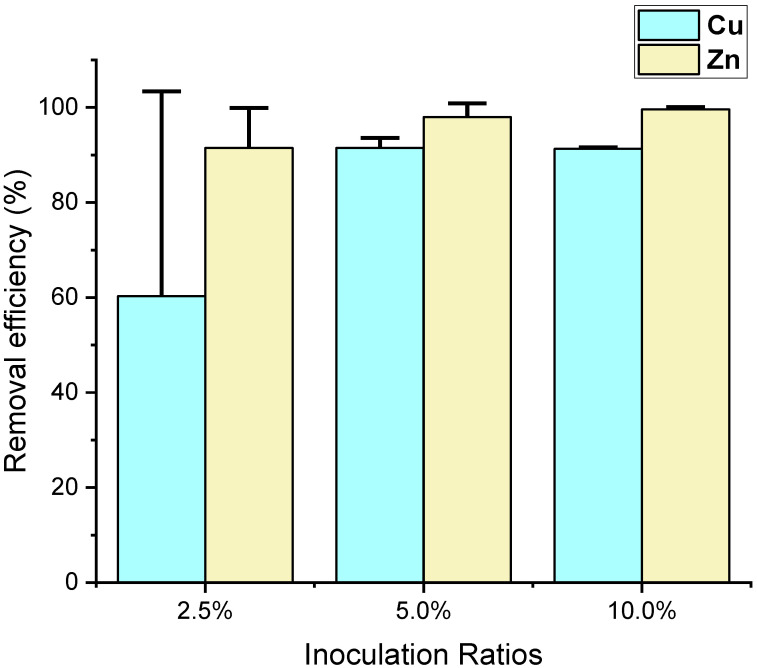
The removal efficiency of copper (Cu) and zinc (Zn) from the filtrated supernatant at pH 5.5, was evaluated at different inoculation ratios using yeast biosorption. Error bars indicate standard deviations.

The inoculation ratios of 2.5%, 5%, and 10% showed copper removal efficiencies of 60.3 ± 43.1%, 91.5 ± 2.1%, and 91.3 ± 0.3%, respectively, indicating no significant difference among them. For zinc biosorption, the groups with 2.5%, 5%, and 10% inoculation ratios showed removal efficiencies of 91.5 ± 8.4%, 98.0 ± 2.9%, and 99.6 ± 0.5%, respectively, also indicating no significant differences among them. Notably, there was a slight increasing trend in zinc removal efficiency with higher inoculum levels (Figure 4 and Table 3). This suggests that at these inoculation ratios, the concentrations of the biosorbent, i.e., yeast, are sufficient, and further increasing the yeast biosorbent amount does not significantly affect the removal efficiency.

Research by Junghans and Straube showed that in the low inoculation ratio group for copper biosorption, a few repeat tests showed no copper adsorption by yeast, possibly due to the biotoxicity of copper ions to microorganisms at low concentrations where yeast was not yet stable [28]. Biotoxicity of copper ions was observed on bacterial strains, such as *Bacillus* spp. [29]. Alternatively, the ionic state of copper ions in the pH 5.5 acetic acid solution might be unstable, requiring further repeat tests and additional analyses to understand the possible causes.

**Figure 5:**
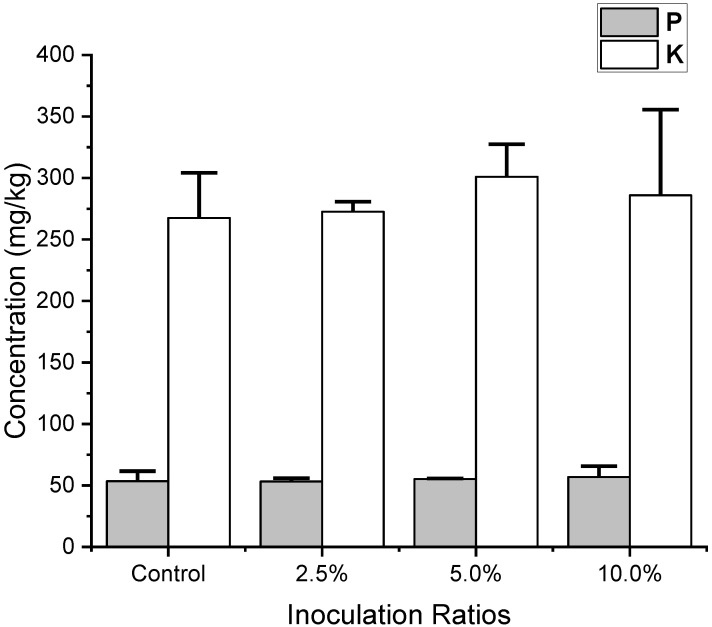
The remaining concentrations of phosphorus (P) and potassium (K) in the filtrated supernatant at pH 5.5 were measured after yeast biosorption at different inoculum ratios. The concentrations are expressed in mg/kg, with phosphorus represented by grey bars and potassium by white bars. Error bars indicate the standard deviation of the measurements.

Results showed no significant differences in phosphorus and potassium concentrations among all experimental groups and the control group (53.5 ± 8.1 mg/kg for phosphorus and 267.5 ± 36.8 mg/kg for potassium). The overall average final potassium concentrations for the inoculation ratios of 2.5%, 5%, and 10% were 272.6 ± 8.0, 301.0 ± 26.4, and 286.0 ± 69.6 mg/kg, respectively, indicating that yeast does not adsorb these two elements during biosorption, resulting in minimal differences among groups and the control group (Figure 5 and Table 3).

Previous studies have shown that increasing the biosorbent amount generally enhances the removal efficiency due to increased chances of contact between heavy metals in wastewater and functional groups on the yeast surface. However, the amount removed per unit weight of yeast decreases [5], as heavy metals not only interact with adsorption sites on the yeast surface at low biosorbent concentrations but also diffuse into cells through concentration gradients [18]. Therefore, the ratio of heavy metal concentration in wastewater to biosorbent amount is one of the primary factors to consider in biosorption experiments.

### 3.3. Factors Affecting Yeast Biosorption

#### 3.3.1. Type of Biosorbent

The yeast used in this experiment was live, and the aim was to determine whether yeast can survive in this type of wastewater and maintain its biosorption properties under these conditions. Previous studies have indicated that dead cells have better biosorption capabilities [5,17,30]. This suggests that even if yeast cannot survive under these conditions, it can still treat the heavy metals in the supernatant. Experimental results showed that although yeast growth was poor at pH 4.5, it still had some capacity to adsorb copper and zinc, with removal efficiencies of 48.6% and 32.9%, respectively (Table 2). At pH 5.5, yeast exhibited better biosorption of copper and zinc, with removal efficiencies of about 48.8% and 97.3%, respectively, and better growth conditions (Table 2). This indicates that adjusting certain factors under these conditions may further enhance yeast growth and enable the collection of by-products like alcohol [9].

#### 3.3.2. Biosorption Time

The biosorption time by yeast for this study was 240 min (Table 1). Most biosorption experiments achieve optimal removal efficiency within a short time, such as within 250 min [23,31]. However, shorter times may only reflect the effects of passive transport. This experiment used live cells to understand the potential role of active transport during the process.

#### 3.3.3. pH Value

pH value is a critical variable in past studies, as it affects yeast growth in solution, the charge of functional groups on the yeast cell wall, the chemical composition of the wastewater solution, and the redox states of heavy metals [18]. The experimental results of this study found that increasing pH from 4.5 to 5.5 significantly enhanced zinc removal, while copper removal efficiency showed little variation within this range (Table 2). This finding aligns with previous research, which indicated that pH has an insignificant impact on copper removal but significantly affects zinc removal at pH 5.5 [21,26].

#### 3.3.4. Incubation Temperature

The incubation temperature for biosorption experiments in this study was maintained at 28 °C to ensure optimal conditions for yeast growth (Table 1). Previous studies have shown that different temperatures (27, 37, 52, and 62 °C) have minimal impact on the adsorption efficiency of dead yeast cells [21]. However, for live cells, temperature must be controlled within specific limits.

#### 3.3.5. Biosorbent Concentration

The average biosorbent, i.e., yeast, concentrations used for this study were 3.59 and 2.06 mg/L for the pH test and yeast concentrations test, respectively (Table 1). After removing refrigerated yeast and activating it for 24 h, the O.D._600_ values ranged from 0.9 to 1.2. In addition to measuring O.D._600_, the dry weights of the yeast were measured to calibrate the yeast concentrations. Differences in biosorbent dosage (inoculum ratio) did not significantly affect removal efficiency. However, some studies indicated that increasing biosorbent dosage at low concentrations significantly enhances removal efficiency [12,25]. Once the removal peak is reached, increasing biosorbent dosage does not improve removal efficiency. High concentrations of yeast may cause aggregation, reducing adsorption efficiency.

By using dead yeast cells, the optimum Cu^2+^ adsorption efficiency occurs at pH = 4, contact time 2 h, 500 mg dried *S. cerevisiae* (from beer waste), initial Cu^2+^ concentration 33,764 mg/L, at room temperature (±28 °C), and shaker speed 150 rpm is 61.4% [21]. To enhance the efficiency of metal biosorption, immobilized brewing yeast on calcium alginate (4 to 6 mm in diameter) was used to extract copper and zinc from metal solutions under both aerobic and anaerobic conditions. Under aerobic conditions, the concentration of zinc ions in an aqueous solution decreased by 99.76% within 2 h, and copper ions by 91.7%, when live yeast was immobilized in calcium alginate [32].

The uptake capacity of lead, cadmium, chromium, copper, cobalt, and zinc increases with higher initial metal concentrations and decreases as biosorbent weight increases. Moreover, some studies showed dead yeast was applied for metal biosorption from metal solutions. The uptake capacity also rises with increasing pH levels, with maximum capacities observed at pH values of 6.0 for lead, 5.5 for cadmium, 2.5 for chromium, 5.5 for copper, 6.0 for cobalt, and 5.5 for zinc. The order of metal ion uptake capacity by yeast is as follows: Pb > Zn > Cr > Co > Cd > Cu. [22]. Some studies suggest that both the source of wastewater and the type of yeast biosorbent (e.g., food industry waste, dried yeast, live yeast, immobilized yeast, or pretreated yeast) significantly influence biosorption efficiency [12]. Therefore, this experiment used yeast strains isolated from rapid-rise baker’s yeast to eliminate other potential interfering factors and examine the utilization and adsorption of copper and zinc by live yeast. While some studies have used simulated wastewater to calculate potential yeast removal efficiency, this experiment employed acid-treated wastewater, containing various metals and ions that might interfere with removal efficiency, leading to varying results.

In summary, this study utilized baker’s yeast to adsorb copper and zinc from the supernatant after acetic sludge extraction. The removal efficiencies achieved were 48.8 ± 25.8% for copper and 97.3 ± 2.9% for zinc at a pH of 5.5 (Table 2). When compared to other studies, the copper removal efficiency (48.8 ± 25.8%) in this research was similar to the efficiency observed in electroplating industrial wastewater with high copper concentrations (61.4% at pH = 4) (Table 4). However, the zinc biosorption efficiency (97.3%) in this study was superior to most other studies listed in Table 4. Notably, the metal acidic extraction using acetic acid and biosorption processes was conducted using environmentally friendly methods, avoiding the use of toxic chemical acids.

## 4. Conclusions

In this study, live yeast was employed as a biosorbent to investigate the biosorption of heavy metals. The experimental results indicated that the pH value had minimal impact on copper biosorption (*p* > 0.05) but significantly influenced zinc biosorption (*p* < 0.05). At a pH of 5.5, yeast was able to adsorb approximately 48.8% of copper and 97.3% of zinc from the acid-extracted supernatant containing heavy metals. The inoculum amount of yeast did not exhibit a significant effect on the biosorption process. Several limitations were identified. Firstly, copper biosorption was less effective, with only about 50% removal, indicating the need for further optimization to enhance copper recovery efficiency. Secondly, the study was conducted on a laboratory scale, and the feasibility of scaling up the process to an industrial level has not been assessed. Lastly, the inoculum amount of yeast did not show a significant effect, suggesting that other variables might play a more critical role and should be investigated further. Future studies should explore alternative biosorbents or modifications to the current yeast biosorbent to improve copper biosorption. Additionally, optimizing conditions like pH and contact time could enhance overall metal recovery. To assess scalability, pilot-scale studies should be conducted, considering economic aspects, practical implementation, and real-world applications. Finally, the residual sludge can be evaluated for use as fertilizer, contributing essential nutrients back to the soil and promoting sustainable agricultural practices.

## Figures and Tables

**Table 1:** Experimental conditions for biosorption of metals from supernatant post-acidic sludge extraction in 125 mL Erlenmeyer Flasks.

Variables	Supernatant/ Diluted Molasses Vol. (mL)	Yeast Concentrations (mg/L)	Inoculation Ratios (%)	pH Values	Incubation Temp./Time/Shaker Speed (°C/h/rpm)
pH Values	45/2.5	3.59	5	4.5, 5.0, 5.5	28/4/100
Inoculation Ratios	45/2.5	2.05	2.5, 5, 10	5.5	28/4/100

**Table 2:** Efficiency of copper (Cu) and zinc (Zn) removal, as well as remaining phosphorus (P) and potassium (K) concentrations at various pH levels following yeast biosorption.

Elements		pH Values			*p*-Value
		4.5	5.0	5.5	
Removal efficiency (%)					
Cu		48.6 ± 4.8	54.9 ± 1.6	48.8 ± 25.8	NS
Zn		32.9 ± 8.1 ^c^	46.7 ± 3.4 ^b^	97.3 ± 2.9 ^a^	<0.05
Concentration (mg/kg)					
P	Initial	72.3	55.6	10.9	
	Final	69.8 ± 8.6	54.3 ± 3.1	10.5 ± 0.4	<0.05
K	Initial	44.7	46.5	40.8	
	Final	43.1 ± 4.7	43.9 ± 2.0	41.4 ± 2.6	NS

Note: NS denotes non-significant differences. Different superscripts (^a^, ^b^, ^c^) within a row indicate significant differences (*p* < 0.05) based on Tukey’s test.

**Table 3:** The efficiency of copper (Cu) and zinc (Zn) removal and remaining concentrations of phosphorus (P) and potassium (K) at various inoculum ratios after yeast biosorption at pH 5.5.

Elements	Control (No Inoculation)	Inoculum Ratio (%)			*p*-Value
		2.5	5.0	10.0	
Removal efficiency (%)					
Cu		60.3 ± 43.1	91.5 ± 2.1	91.3 ± 0.3	NS
Zn		91.5 ± 8.4	98.0 ± 2.9	99.6 ± 0.5	NS
Concentration (mg/kg)					
P	53.5 ± 8.1	53.3 ± 2.6	55.2 ± 0.6	56.9 ± 8.7	NS
K	267.5 ± 36.8	272.6 ± 8.0	301.0 ± 26.4	286.0 ± 69.6	NS

Note: NS denotes non-significant differences.

**Table 4:** The efficiency of metal biosorption by various types of yeast includes details such as the type of metal ions, pH values, yeast condition, yeast source/biosorption conditions, initial metal concentrations/sources, removal efficiency percentages, and references.

Metals	pH Values	Yeast Condition	Yeast Source/ Biosorption Conditions	Initial Metal Concentrations/ Sources of Metals	Removal (%)	References
Cr (VI)	5, 7, 9	Dead (dry at 60 °C, 24 h)	Dried cell (25 °C, 3 h)	100 mg/L (Potassium dichromate solution)	Cr: 99.66 (pH = 5)	[13]
Cu (II)	2, 3, 4, 5, 6	Dead (dry at 80 °C, 28 h)	Beer waste (28 °C, 2 h)	33,746 mg/L (Electroplating industry waste)	Cu: 61.40 (pH = 4)	[26]
Pb, Zn, Cr, Co, Cd, Cu	2, 3, 4, 5.5, 6, 8	Dead (dry at 60 °C, 6 h)	Raw yeast (25 °C, 0.5 h)	100 mg/L (Metal solutions)	Pb > Zn > Cr > Co > Cd > Cu (pH = 5.5)	[21]
Cd (II), Zn (II), Cu (II)	3, 4.5, 6, 7, 8.5, 9.5, 11	Living	Baker’s yeast (25 °C, 0.5 h)	5.0 mg/L (Metal solutions)	Cu: 55.0 (pH = 6) Zn: 69.8 (pH = 6) Cd: 95.6 (pH = 8.5)	[25]
Cu (II), Zn (II)	4.5, 5.0, 5.5	Living	Baker’s yeast (28 °C, 4 h)	Cu: 4.01 mg/kg Zn: 43.7 mg/kg (Supernatant after acidic sludge extraction)	Cu: 48.8 (pH = 5.5) Zn: 97.3 (pH = 5.5)	This study

## Data Availability

Data are contained within the article.
